# Combination of Youhua Kuijie Prescription and sulfasalazine can alleviate experimental colitis via IL-6/JAK2/STAT3 pathway

**DOI:** 10.3389/fphar.2024.1437503

**Published:** 2024-09-10

**Authors:** Lili Tang, Yuedong Liu, Hongwu Tao, Wenzhe Feng, Cong Ren, Yuping Shu, Ruijuan Luo, Xiangyi Wang

**Affiliations:** ^1^ Liaoning University Of Traditional Chinese Medicine, Shenyang, China; ^2^ The Third Affiliated Hospital of Liaoning University Of Traditional Chinese Medicine, Shenyang, China; ^3^ The Second Affiliated Hospital of Liaoning University Of Traditional Chinese Medicine, Shenyang, China; ^4^ Affiliated Hospital of Shaanxi University of Chinese Medicine, Xi’an, China; ^5^ Fujian People’s Hospital, Fuzhou, China; ^6^ Kaifeng Traditional Chinese Medicine Hospital, Kaifeng, China

**Keywords:** Youhua Kuijie prescription, ulcerative colitis, animal experiment, network pharmacology, molecular mechanism

## Abstract

**Introduction:**

Youhua Kuijie prescription (YHKJ) is a hospital preparation that is composed of nine kinds of herbs. Sulfasalazine (SASP) is widely used as a first-line clinical treatment for UC. Traditional Chinese medicine and Western medicine have their own advantages in the treatment of UC, and the mechanism of YHKJ combined with SASP in the treatment of UC needs to be investigated.

**Methods:**

In this study, the therapeutic mechanism of YHKJ combined with SASP in the treatment of UC was predicted by network pharmacology and molecular docking. The chemical components and related targets of YHKJ were obtained from the TCMSP database. The chemical structure of SASP was obtained from the PubChem server, and related targets of SASP molecules were identified using the PharmMapper database. UC-related targets were obtained from the DisGeNET, GeneCards, OMIM, TTD, DrugBank and PharmGkb databases.

**Results:**

In total, 197 shared targets were identified by constructing a Venn diagram. PPI network data obtained from the STRING database were imported into Cytoscape to visualize the “drug-disease” target network, and STAT3 was selected as the core target by topological analysis. Gene Ontology revealed the biological functions of target genes, and KEGG analysis revealed that the core target STAT3 was differentially expressed in Th17 cells and the JAK-STAT signaling pathway. Thus, the core target STAT3 was subjected to molecular docking with the top 10 components, including nine YHKJ components (quercetin, luteolin, ursolic acid, daidzein, kaempferol, wogonin, myricetin, formononetin, indirubin) and SASP (C18H14N4O5S). The molecular docking results showed that STAT3 had favorable binding with the nine YHKJ components and SASP; STAT3 had the strongest binding with ursolic acid (−10.26 kcal/mol), followed by SASP (−8.54 kcal/mol). Qualitative analysis of the chemical constituents of YHKJ by HPLC revealed that sitosterol, ursolic acid, myricetin, daidzein, quercetin, kaempferol and formononetin were the main components. Additional experiments verified that YHKJ combined with SASP inhibited activation of the IL-6/JAK2/STAT3 pathway and alleviated inflammation in UC model rats.

**Discussion:**

Our results showed that seven chemical components in YHKJ cooperate with SASP to interfere with activation of the IL-6/JAK2/STAT3 pathway, thus playing a role in the treatment of UC.

## 1 Introduction

Ulcerative colitis (UC) is an inflammatory disease of the colon, that has a high incidence in Europe and America (Anyane-Yeboa et al., 2023; Lo et al., 2023). In recent years, the incidence and canceration rate of UC in Asia and Africa have increased, and UC has been listed as a globally recognized refractory disease (Kim et al., 2020; Sham et al., 2018). The etiology of UC is not clear, and research suggests that it may be related to heredity, environment, immunity, infection, etc (Kobayashi et al., 2020; Li et al., 2024). Because of the complexity of the pathogenesis of UC, clinical treatment is also more difficult. Identifying a safe, low-cost treatment strategy with few side effects that can effectively inhibit immune inflammatory reactions and reduce the rate of recurrence rate is challenging.

Traditional Chinese medicine (TCM) can be used to treat irritable bowel syndrome, UC and many other diarrhea-related diseases, and TCM has the advantages of low cost, few side effects, and limited drug resistance. Youhua Kuijie Prescription (YHKJ) is an in-hospital preparation that is used at the Third Affiliated Hospital of Liaoning University of Traditional Chinese Medicine. YHKJ was prepared from Atractylodes Macrocephala Koidz, Herba Patriniae, Hedysarum Multijugum Maxim., Indigo Naturalis, Carthami Flos, Picrorhizae Rhizoma, Pulsatilliae Radix, Atractylodes Lancea (Thunb.) Dc., and Angelica sinensis Radix, which are nine TCMs that are effective for the clinical treatment of UC, Table 1. YHKJ has been used in the clinic for more than 10 years and have unique characteristics and advantages in treatment (Wang, 2023). Clinical studies revealed that YHKJ can effectively alleviate the clinical symptoms of UC patients, reduce the expression of CRP and TNF-α and promote intestinal mucosal repair, and the effective rate can reach 90.0% (Wang, 2023; Wang et al., 2022). Animal experiments and cell experiment also confirmed that YHKJ can reduce the expression of IL-1β and IL-6, increase the content of the epidermal growth factor EGF and promote healing of the intestinal mucosa in model rats with TNBS-induced UC. As a 5-aminosalicylic acid preparation, sulfasalazine (SASP) is a first-line drug that is used for the treatment of UC and can promote UC remission; SASP has the advantages of a definite curative effect and low cost (Shin et al., 2020; Sutherland and Macdonald, 2006).

**TABLE 1:** Composition of Youhua Kuijie prescription.

Chinese pinyin name	Latin name	Family	Genus	Part used
Baizhu	Atractylodes Macrocephala Koidz	Compositae	Atractylodes	Root
Baijiangcao	Herba Patriniae	Valerianaceae	Patrinia	The whole herb
Huangqi	Hedysarum Multijugum Maxim	Leguminosae	Astragalus L	Root
Qingdai	Indigo Naturalis	Brassicaceae	Isatis L	Stem and leaf
Honghua	Carthami Flos	Compositae	Carthamus L	Flower
Huhuanglian	Picrorhizae Rhizoma	Plantaginaceae Juss	Coptis Salisb	Root
Baitouweng	Pulsatilliae Radix	Ranunculaceae Juss	Pulsatilla Adans	Root
Cangzhu	Atractylodes Lancea (Thunb.)Dc	Compositae	Atractylodes	Root
Danggui	Angelicae Sinensis Radix	Apiaceae	Angelica L	Root

**FIGURE 1:**
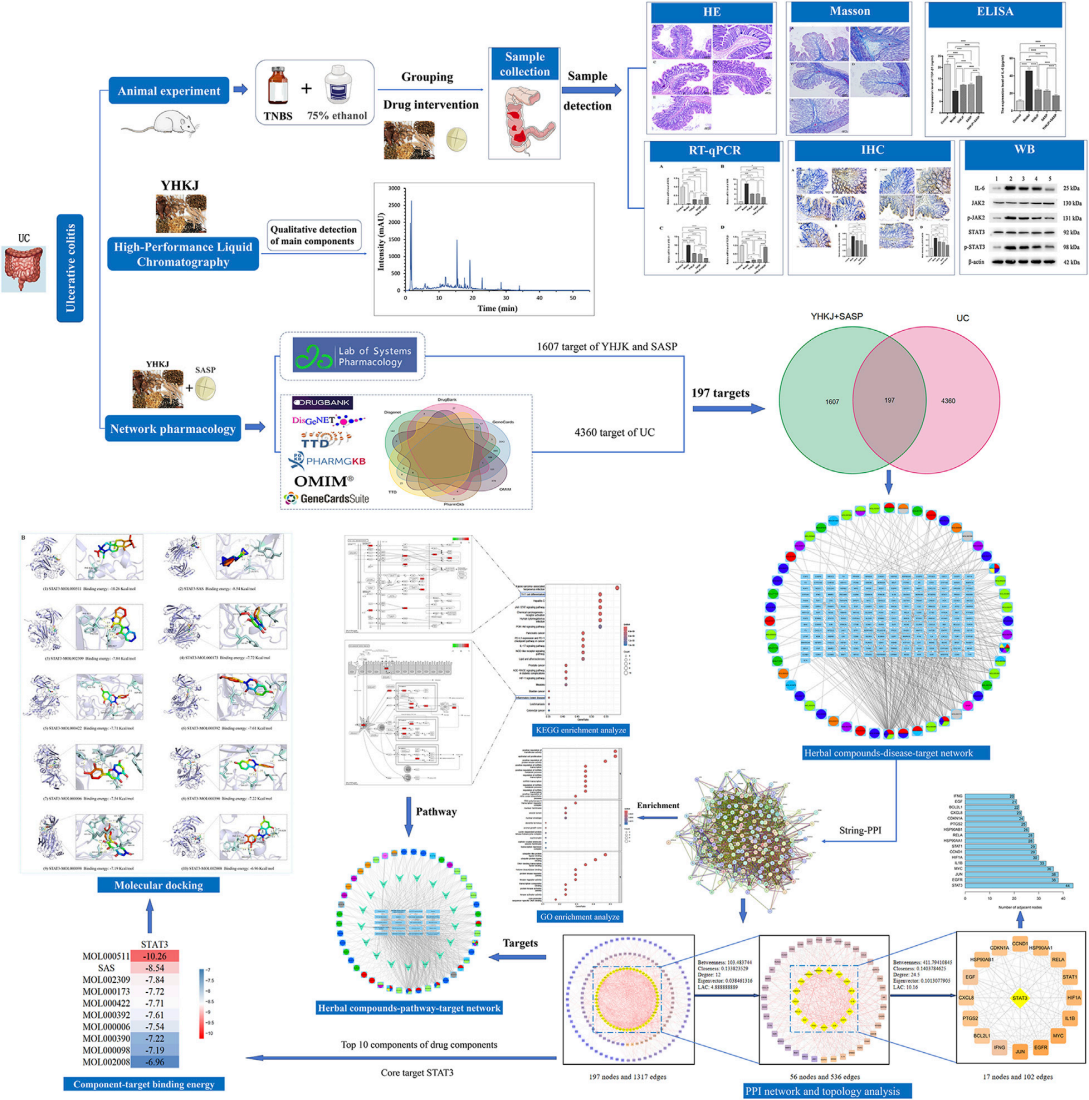
The general flow chart.

Therefore, in this study, the hospital preparation YHKJ was combined with SASP as the treatment, and the chemical components of YHKJ and SASP and their targets and pathways involved in UC were predicted and analyzed by network pharmacology. The binding between the chemical components of YHKJ and SASP and UC disease targets were predicted by molecular docking, and the molecular mechanism of YHKJ combined with SASP in treating UC was systematically analyzed. Animal experiments were used to verify the underlying molecular mechanism, and the mechanism by which YHKJ combined with SASP alleviates UC was investigated via bioinformatics and basic research, the general flow chart of this study is shown in Figure 1.

## 2 Materials and methods

### 2.1 Network pharmacology and molecular docking

#### 2.1.1 Screening the effective components of YHKJ and SASP and their targets

The chemical constituents of 9 herbs contained in YHKJ were searched in the TCMSP database, and the active components and related targets of the drugs were screened using oral bioavailability (OB) ≥ 30% and drug-like properties (DL) ≥ 0.18 as pharmacokinetic parameters. The chemical structure of SASP was obtained from the PubChem server, and the PharmMapper server was used to identify the potential targets of small molecules (drugs, natural products, etc.). Molecular structure analysis was carried out by using the PharmMapper database to screen SASP-related targets.

#### 2.1.2 Acquisition and screening of UC-related targets

The disease targets of UC were obtained by searching the DisGeNET, Genecard, OMIM, TTD, DrugBank and PharmGkb databases. The shared drug targets of YHKJ combined with SASP and UC disease targets were identified and imported into Cytoscape software to construct a “drug-disease-target” visual network diagram, and the Venn 2.1.0 platform was used to construct Venn diagram.

#### 2.1.3 Construction of the protein-protein interaction (PPI) and the identification of core targets

The shared targets were imported into the STRING database, “*Homo sapiens*” and “highest confidence (0.9)” were defined, and a PPI network was constructed. The relevant PPI data were imported into Cytoscape 3.7.1 software for visual analysis. Targets with values higher than the median values of degree, closeness, betweenness, eigenvector and local average connectivity were selected as key targets, and the key targets with the most corresponding targets were selected as core targets.

#### 2.1.4 Gene Ontology (GO) and kyoto encyclopedia of genes and genomes (KEGG) enrichment analyses

Using R 4.3.2 software, the key targets of YHKF and SASP in treating UC were analyzed by GO and KEGG enrichment analyses with the “clusterProfiler” program, and the significant enrichment results were identified and plotted with “enrichplot” and “ggplot2”.

#### 2.1.5 Molecular docking

The core targets were used as protein receptors, and the molecular structures of the protein receptors were downloaded from the RCSB PDB database. According to the degree value, the top 10 chemical components of YHKJ combined with SASP were selected as small molecular ligands, and the structures of the SDF-format compounds of the small molecular ligands were obtained from the PubChem database, which was converted into mol2 format by Open Babel 2.4.1 software. AutoDock Vina software was used for molecular docking, the binding strength between the core target and chemical components of YHKJ combined with SASP was predicted, and the binding energy results were extracted. R Studio was used to construct the binding energy thermogram, and PyMol 4.6.0 software was used to construct the overall and local molecular docking diagrams.

### 2.2 Qualitative analysis of the main components of YHKJ and verification in animal experiments

#### 2.2.1 Drugs and reagents

YHKJ granules were purchased from the Pharmacy of the Third Affiliated Hospital of Liaoning University of Traditional Chinese Medicine, with the production number 0068030268, and the refining of TCM granules was performed by Sichuan Xinlv Pharmaceutical Technology Development Co., Ltd. Standard: sitosterol (>98%, CAS: 83-46-5), ursolic acid (>98%, CAS: 77-52-1), myricetin (>98%, CAS: 529-44-2), daidzein (>98%, CAS: 486-66-8), quercetin (>98%, CAS: 117-39-5), kaempferol (>98%, CAS: 520-18-3), and formononetin (>98%, CAS: 485-72-3) was purchased from Shanghai Yuanye Biotechnology Co., Ltd. And 2,4,6-trinitrobenzene sulfonic acid (TNBS) was purchased from Beijing Sihai Longxing Technology Co. Ltd. (CAS:2508-19-2). Masson tricolor dye solution (Fuzhou Maixin Biotechnology Development Co., Ltd., item number: MST-8003) was used. Rat IL-6 and rat TGF-β1 ELISA kits were used (Shanghai Enzyme Linked Biotechnology Co., Ltd.; item numbers: ML064292-Z and ML00285 6-Z). Anti-IL-6 (Abcam, ab9324), recombinant anti-JAK2 (Abcam, ab32101), anti-phospho-JAK2 (Beijing Biosynthesis Biotechnology Co.,Ltd., bs-2485R), recombinant anti-STAT3 (phospho S727) (Abcam, ab32143), and recombinant anti-STAT3 (Abcam, ab68153) antibodies were used.

#### 2.2.2 Qualitative analysis of the main pharmaceutical components of YHKJ by HPLC

Because YHKJ is a complex TCM, an Agilent HPLC-DAD instrument was used for qualitative detection by HPLC to identify the main chemical components of YHKJ and evaluate drug quality. Standard samples (0.024 g quercetin, 0.015 g ursolic acid, 0.011 g kaempferol, 0.02 g sitosterol, 0.016 g daidzein, 0.021 g myricetin, and 0.019 g formononetin) were placed in a 20 mL flask, and 10 mL methanol was added. Three grams of YHKJ granules were put into a 50 mL beaker, and 20 mL of methanol was added for ultrasonic treatment (working rate: 200 W; frequency: 37 kHz) for 1 h. The detection conditions were as follows: chromatographic column, 4.6 mm × 250 mm, 5 μm; mobile phase, acetonitrile (A)-0.1% phosphoric acid solution (B); column temperature, 30°C; detection wavelength, 210 nm; sample volume, 10 μL; flow rate, 1.0 mL/min; and gradient elution procedure, 0∼10 min (10%→25% A), 10∼30 min (25%→45% A), 30∼35 min (45%→80% A), and 35∼55 min (80%→100% A).

#### 2.2.3 Animal experiments

##### 2.2.3.1 Preparation of drug solution

YHKJ granules were added to purified water to prepare YHKJ solution at a concentration of 0.738 g/mL, and SASP tablets were dissolved in purified water to obtain SASP solution at a concentration of 0.018 g/mL.

##### 2.2.3.2 Experimental animals

Male Sprague Dawley (SD) rats (200–240 g) were obtained from Liaoning Chang sheng Biotechnology Co., Ltd., and the license number was SCXK (Liao) 2020-0001. Rats were kept in the animal room at Liaoning University of Traditional Chinese Medicine. This animal experiment was approved by the Experimental Animal Ethics Committee of Liaoning University of Traditional Chinese Medicine (2100004202101, approved on 27 October 2022).

##### 2.2.3.3 Grouping of experimental animals and establishment of the UC model

SD rats were randomly divided into a control group, a model group, a YHKJ group, a SASP group and a YHKJ + SASP group, with 12 rats in each group. The UC model was established with TNBS. UC model rats were utilized in the model and treatment groups. Anhydrous ethanol was diluted to 50%, TNBS and 50% ethanol were mixed at a ratio of 1:2 to prepare the enema solution, and 30 mg/kg enemas (8 cm away from the anus) were applied to the rats to establish the UC model (Seto et al., 2017; Zeng et al., 2017; Zeng et al., 2018; Zhang et al., 2021). After successful establishment of the model, the control group and model group were given 3 mL of sterilized water for injection, the YHKJ group was given YHKJ solution (crude drug concentration of 11.07 g/kg), the SASP group was given SASP solution (crude drug concentration of 0.27 g/kg), and the YHKJ + SASP group was given YHKJ solution combined with SASP solution (Wei et al., 2010). After 14 days of intervention, the experimental rats were sacrificed, and samples were taken.

##### 2.2.3.4 Colon pathology and fibrosis evaluation

The collected colon tissues were stored in 4% paraformaldehyde, dehydrated in ethanol, cleared in xylene, and then immersed in liquid paraffin for embedding and fixation. After cooling and molding, the wax block was cut into 3–5 µm thick tissue sections, and the tissue sections were stained with hematoxylin and eosin (HE) and Masson dye to observe the pathology and fibrosis of the colon.

##### 2.2.3.5 Enzyme-linked immunosorbent assay

To observe the effects of YHKJ on serum inflammatory factors and anti-inflammatory factors in UC model rats, we collected samples from UC model rats and measured the expression of IL-6 and TGF-β1 using an ELISA kit (Shanghai Enzyme Linked Biotechnology Co., Ltd., Shanghai, China).

##### 2.2.3.6 Reverse transcription‒quantitative PCR (RT‒qPCR)

Five mUnited Statesilligrams of colon tissue was collected, RNA was extracted with a total RNA isolation kit (Vazyme, Nanjing, China), and cDNA was prepared with RT Master Mix (ABclonal, United States). The following primers were used: TGF-β1 forward primer 5′-AACAACGC AATCTATGACAAAACC-3′ and reverse primer 5′-TACCAAGGTAACGCCA GGAATT-3'; IL-17A forward primer 5′-GCC​CTG​CTG​TCT​GCT​A-3′ and reverse primer 5′-TGG​ACG​GTG​GTT​GGT​TG CTGA-3'; Foxp3 forward primer 5′-GTGTAGATGCAG ACCCTCGTACA-3′ and reverse primer 5′-TGG​GGT​TAG​TGG​CAA​GTG​ATA​C-3'; RORγt forward primer 5′-TCGGTCTCCTATCCCC ATTA-3 and reverse primer 5′-TCGGTCTCCTATCCC CATTA-3'. The qPCR was performed with SYBR Green (ABclonal, United States) (diluted with deionized water) on an automatic fluorescence quantitative PCR instrument (Bio-Rad, United States), and the relative mRNA level was calculated by the 2^−ΔΔCT^ method.

##### 2.2.3.7 Immunohistochemistry (IHC)

Paraffin-embedded tissue sections were baked, dewaxed, rehydrated, and then heat-repaired with high-pressure antigen. Hydrogen peroxide (3%) was added dropwise for 10 min to block endogenous peroxidase activity. After the addition of goat serum for 15 min, the primary antibody (p-JAK2/p-STAT3) was added, and the samples were incubated overnight at 4°C. A biotin-labeled secondary antibody was added, and the samples were incubated at 37°C for 30 min and then incubated with peroxidase-coupled streptavidin for 10 min. The sections were stained with 3,3′-diaminobenzidine (DAB, Solaibao, item number DAitem1016), counterstained with hematoxylin, dehydrated with gradient ethanol and xylene, and sealed with neutral gum. The average optical density (AOD) was calculated by Image-Pro Plus 5.1 (United States).

##### 2.2.3.8 Western blotting (WB)

Fifteen milligrams of colon tissue was mixed with liquid nitrogen and ground thoroughly. Then, the protein was extracted with RIPA lysis buffer (Biyuntian, P0013C), and the protein concentration was analyzed using the BCA kit (Biyuntian, P0012). Proteins were separated by SDS‒PAGE, and the transfer time in transfer buffer was adjusted according to the molecular weight so that proteins were transferred to 0.22 µm PVDF membranes. The membrane was soaked in 5% skim milk and incubated for 1 h. After removing the sealing liquid, primary antibody was added, and the samples were placed on a shaker at 4°C overnight. Washed three times with TBST, the secondary antibody was added and incubated for 1 h. After the membranes were washed with TBST, enhanced chemiluminescence (ECL) reagent was used to detect protein expression. The images were collected by an automatic chemiluminescence image analysis system (Shanghai Tanon).

#### 2.2.4 Statistical analysis

GraphPad Prism (version 10.1, GraphPad, United States) was used to analyze the data, and the results are expressed as the mean ± standard deviation. The differences between groups were analyzed by one-way analysis of variance. When the result was *p < 0.05, there was a significant difference, and the significance of the difference increases gradually as follows: **p < 0.01, ***p < 0.001, and *****p < 0.0001.

## 3 Network pharmacology analysis results

### 3.1 Chemical constituents of YHKJ and SASP- and UC-related targets

**FIGURE 2:**
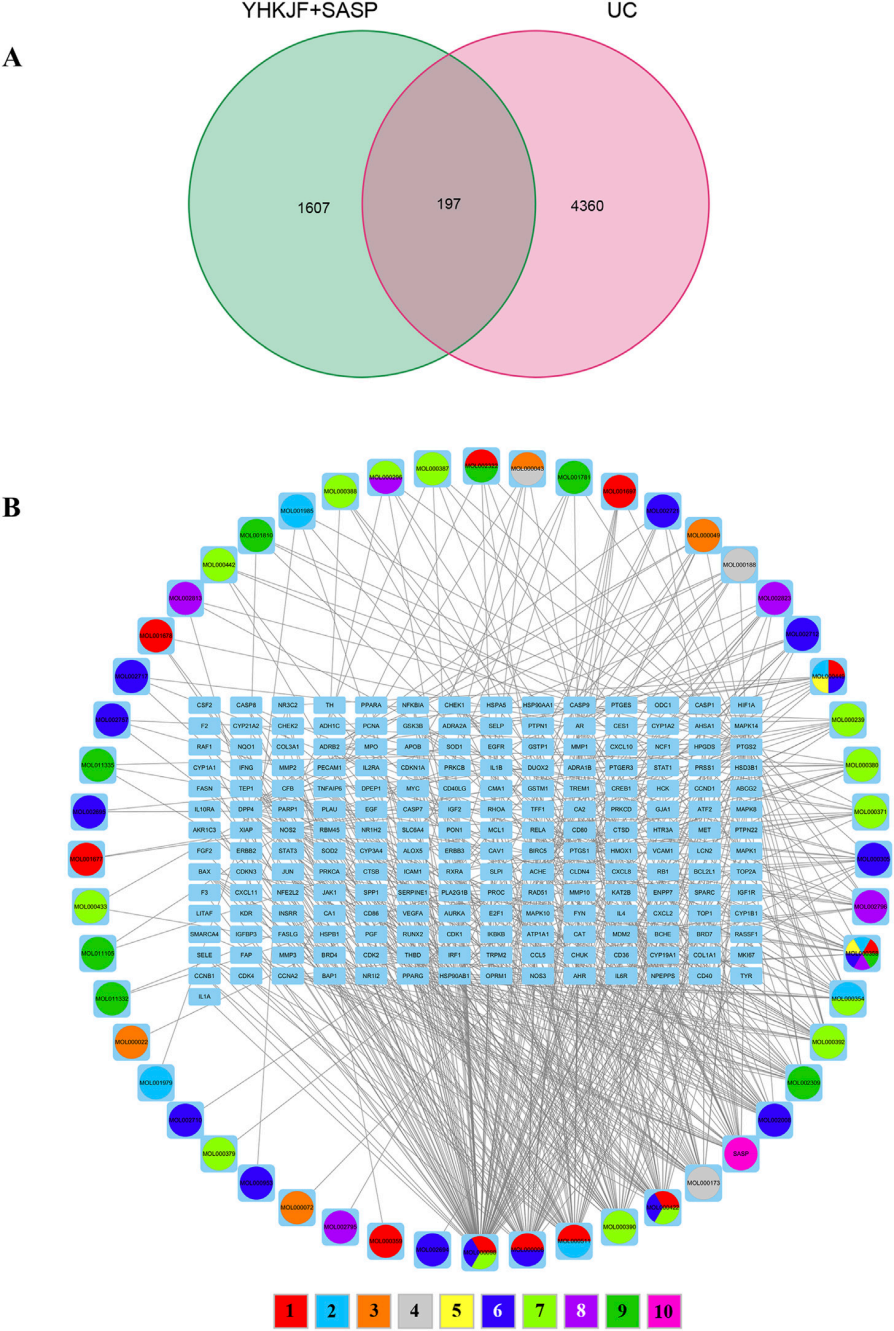
Correlation analysis between drug targets and disease targets. **(A)** Venn diagram of intersection targets between YHKJ + SASP and UC; **(B)** visualization network diagram of the intersection targets of YHKJ + SASP and UC; 1 Baijiangcao, 2 Baitouweng, 3 Baizhu, 4 Cangzhu, 5 Danggui, 6 Honghua, 7 Huangqi, 8 Huhuanglian, 9 Qingdai, 10 SASP.

A total of 105 chemical constituents of YHKJ were identified, including 7 Herba Patriniae, 13 Pulsatilliae Radix, 5 Atractylodes Macrocephala Koidz, 18 Atractylodes Lancea (Thunb.) Dc, 13 Angelicae Sinensis Radix, 5 Carthami Flos, 18 Hedysarum Multijugum Maxim, 13 Picrorhizae Rhizoma, and 5 Indigo Naturalis, with a total of 1,563 corresponding targets. There were 44 targets related to SASP components. A total of 4,360 disease targets corresponding to UC were found through 6 disease databases, Supplementary Image S1. The drug targets of YHKJ and SASP and the disease targets of UC were introduced into the Venn 2.1.0 platform, a total of 197 shared targets were obtained, and a Venn diagram was constructed, Figure 2A. The drug composition and shared targets of YHKJ and SASP were imported into Cytoscape 3.9.1 software for analysis, and a visual network diagram of “drug-disease-targets” was constructed, Figure 2B. The analysis results showed that quercetin (MOL000098), a common component of Herba Patriniae, Carthami Flos and Hedysarum Multijugum Maxim., had the largest number of targets, with a total of 102 targets. Luteolin (MOL000006), a common component of Herba Patriniae and Carthami Flos, has 45 targets. There were 29 targets corresponding to SASP Table 2.

**TABLE 2:** Key herbal ingredient and the number of corresponding targets.

Name/Chemical formula	MOL ID	Corresponding composition	OB (%)	DL	Number of targets
quercetin	MOL000098	Baijiangcao/Honghua/Huangqi	46.43	0.28	102
luteolin	MOL000006	Baijiangcao/Honghua	36.16	0.25	45
ursolic acid	MOL000511	Baitouweng/Baijiangcao	16.77	0.75	42
daidzein	MOL000390	Huangqi	19.44	0.19	39
kaempferol	MOL000422	Baijiangcao/Honghua/Huangqi	41.88	0.24	36
wogonin	MOL000173	Cangzhu	30.68	0.23	30
C18H14N4O5S	NR	SASP	NR	NR	29
myricetin	MOL002008	Honghua	13.75	0.31	25
formononetin	MOL000392	Huangqi	69.67	0.21	21
indirubin	MOL002309	Qingdai	48.59	0.26	21

Note: OB, oral bioavailability; DL, drug-like properties; NR, not reported.

### 3.2 PPI network construction and core target identification

**FIGURE 3:**
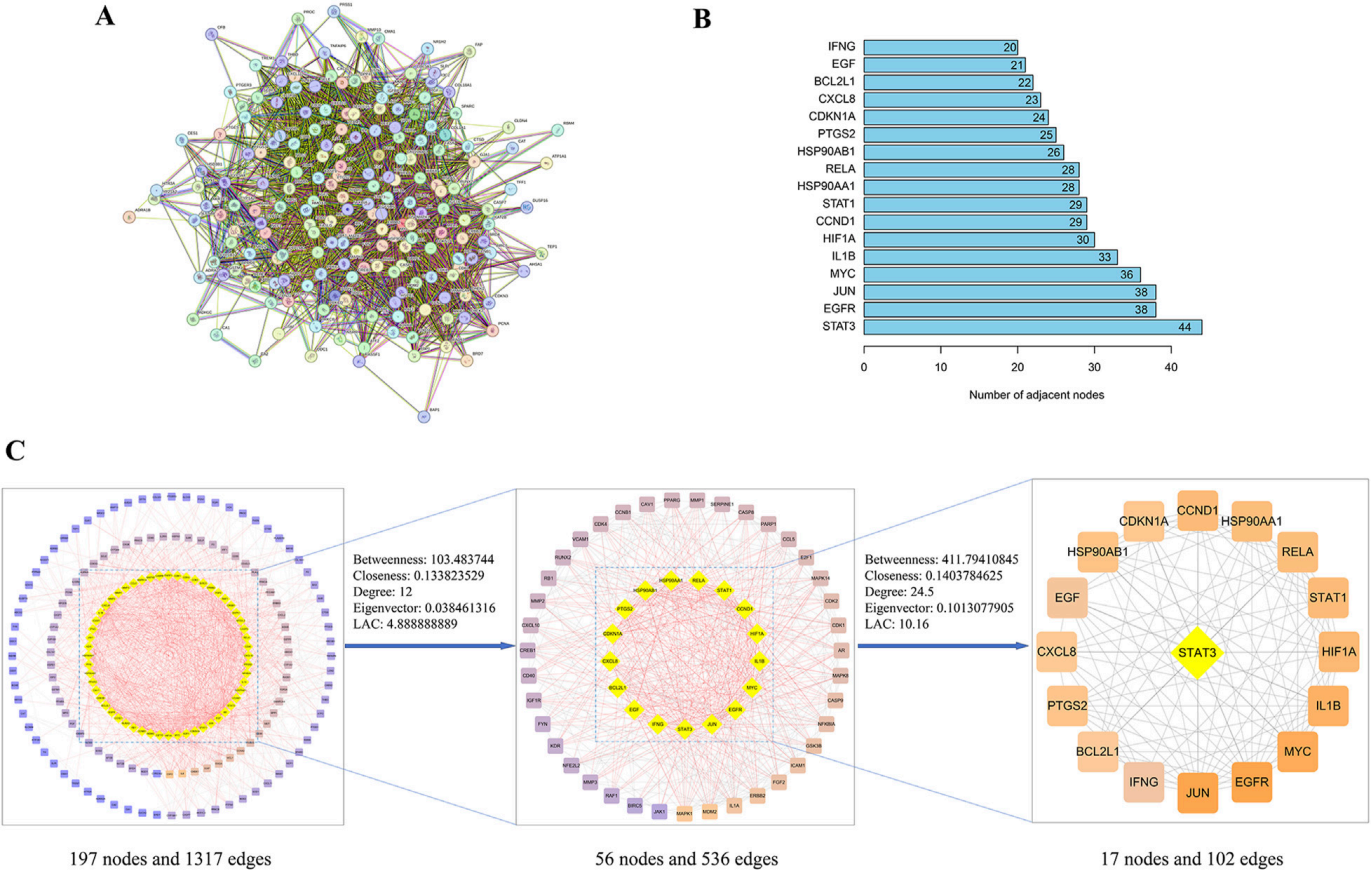
PPI network and key target screening. **(A)** PPI network diagram; **(B)** flow chart of key targets screening in the PPI network; **(C)** top 17 targets and corresponding degree values.

We imported 197 intersecting targets into the String database to construct a PPI network. Network analysis revealed 197 nodes and 1,317 protein interactions, with an average node degree of 13.4, Figure 3A. The PPI network data were imported into Cytoscape for visualization and topological analysis of the “drug-disease” target network. After screening for targets whose degree, closeness, betweenness, eigenvector and local average connectivity were greater than the median values, 17 key targets, including STAT3, EGFR, JUN, MYC, IL1B, HIF1A, CCND1, STAT1, RELA, HSP90AA1, HSP90AB1, PTGS2, and CDKN1A, were identified, Figure 3B, Table 3. We speculate that YHKJ and SASP may play synergistic therapeutic roles through these 17 key targets; among the targets, STAT3 had the largest number of corresponding targets, with a total of 44 targets, Figure 3C. Thus, STAT3 was selected as the core target for subsequent molecular docking and experimental studies.

**TABLE 3:** Topological score of key targets.

Name	Betweenness	Closeness	Degree	Eigenvector	LAC
STAT3	302.2558652	0.833333333	44	0.256566882	15.95454545
EGFR	188.2908494	0.763888889	38	0.2333235	15
JUN	162.2961842	0.763888889	38	0.241509199	16.31578947
MYC	133.5845134	0.743243243	36	0.222588494	15.61111111
IL1B	115.329795	0.714285714	33	0.20133093	14.54545455
HIF1A	82.05545782	0.6875	30	0.194912553	13.26666667
CCND1	67.55248011	0.679012346	29	0.185118765	14.48275862
STAT1	91.42234214	0.679012346	29	0.185630053	13.03448276
RELA	79.19973195	0.670731707	28	0.184223786	13
HSP90AA1	69.52500193	0.670731707	28	0.181013584	13.21428571
HSP90AB1	61.63084992	0.654761905	26	0.168600813	12.15384615
PTGS2	52.69804701	0.647058824	25	0.159129694	12.24
CDKN1A	59.72528166	0.639534884	24	0.157129258	12.16666667
CXCL8	35.98724716	0.625	23	0.143987074	13.13043478
BCL2L1	32.42837425	0.625	22	0.150561318	11.63636364
EGF	34.16226028	0.617977528	21	0.142841339	10.57142857
IFNG	27.43490033	0.611111111	20	0.119762778	11.8

Note: LAC, local average connectivity.

### 3.3 GO enrichment analysis

**FIGURE 4:**
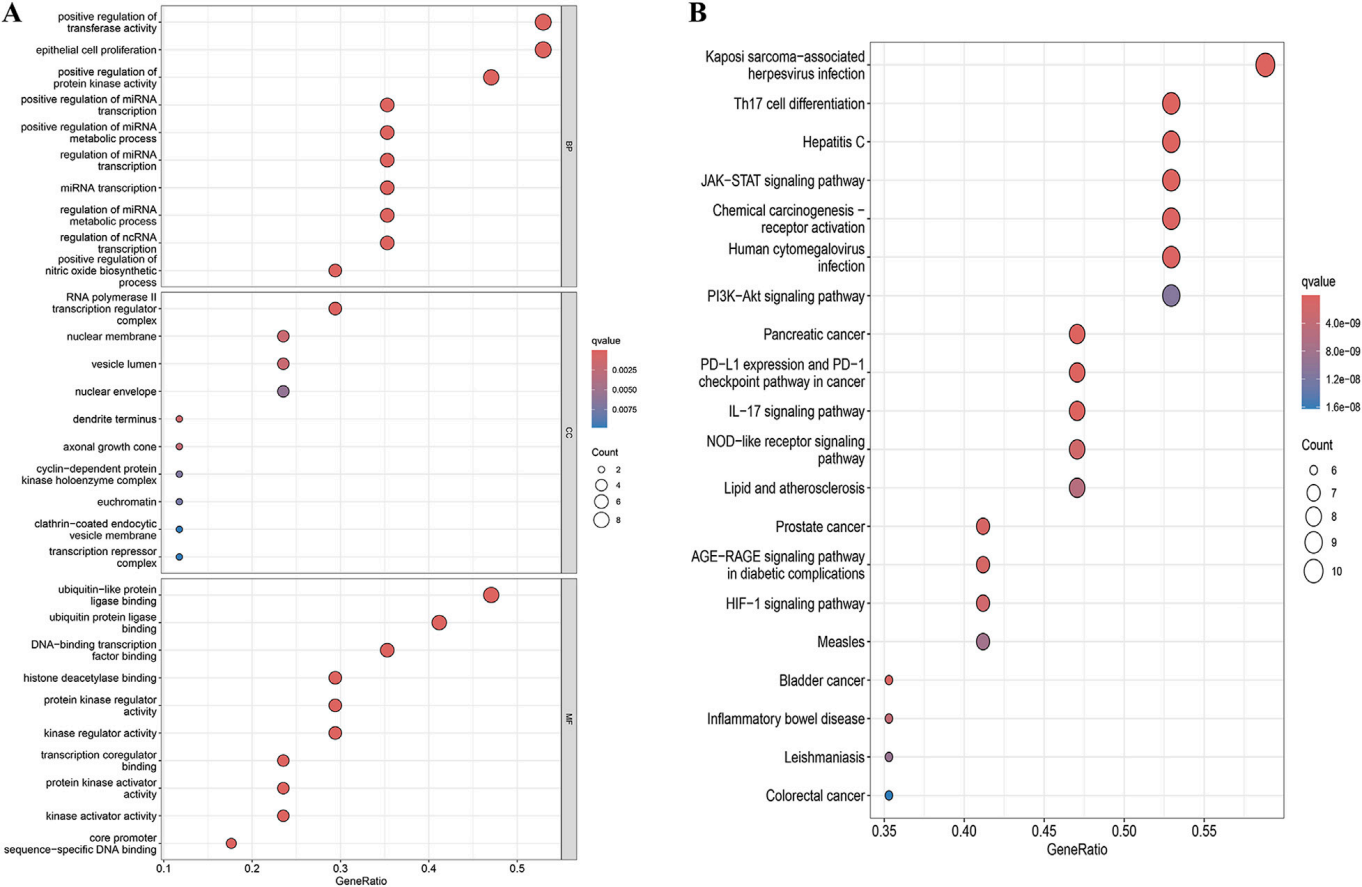
GO and KEGG enrichment analysis results. **(A)** GO enrichment analysis; **(B)** KEGG enrichment analysis.

We obtained 1,346 GO enrichment results for YHKJ and SASP targets in the treatment of UC, including 1,224 biological process (BP), 47 cellular component (CC) and 75 molecular function (MF) terms. The biological processes with enrichment of YHKJ and SASP targets in the treatment of UC were mainly related to positive regulation of transferase activity and protein kinase activity, regulation of miRNA transcription and metabolism, and positive regulation of nitric oxide biosynthesis. The cell components involved included the RNA polymerase II transcription regulatory complex, nuclear membrane, vesicle lumen,and euchromatin. The molecular functions included ubiquitin and ubiquitin-like protein ligase binding, protein kinase activity, and kinase regulatory activity, Figure 4A.

### 3.4 KEGG enrichment analysis and drug-pathway-target network visualization

We identified 119 KEGG pathways involved in YHKJ and SASP treatment of UC, including Th17 cell differentiation, IL-17, JAK-STAT, AGE-RAGE, HIF-1, NOD-like receptor, inflammatory bowel disease, and the PI3K-Akt signaling pathway. These findings suggest that YHKJ and SASP play a role in treating UC through multiple mechanisms, Figure 4B. The drug components, related signaling pathways and 17 key targets were imported into Cytoscape software for drug-pathway-target network analysis and visualization, Supplementary Image S2. We found that YHKJ and SASP components interacted with multiple signaling pathways and key targets.

### 3.5 Molecular docking

**FIGURE 5:**
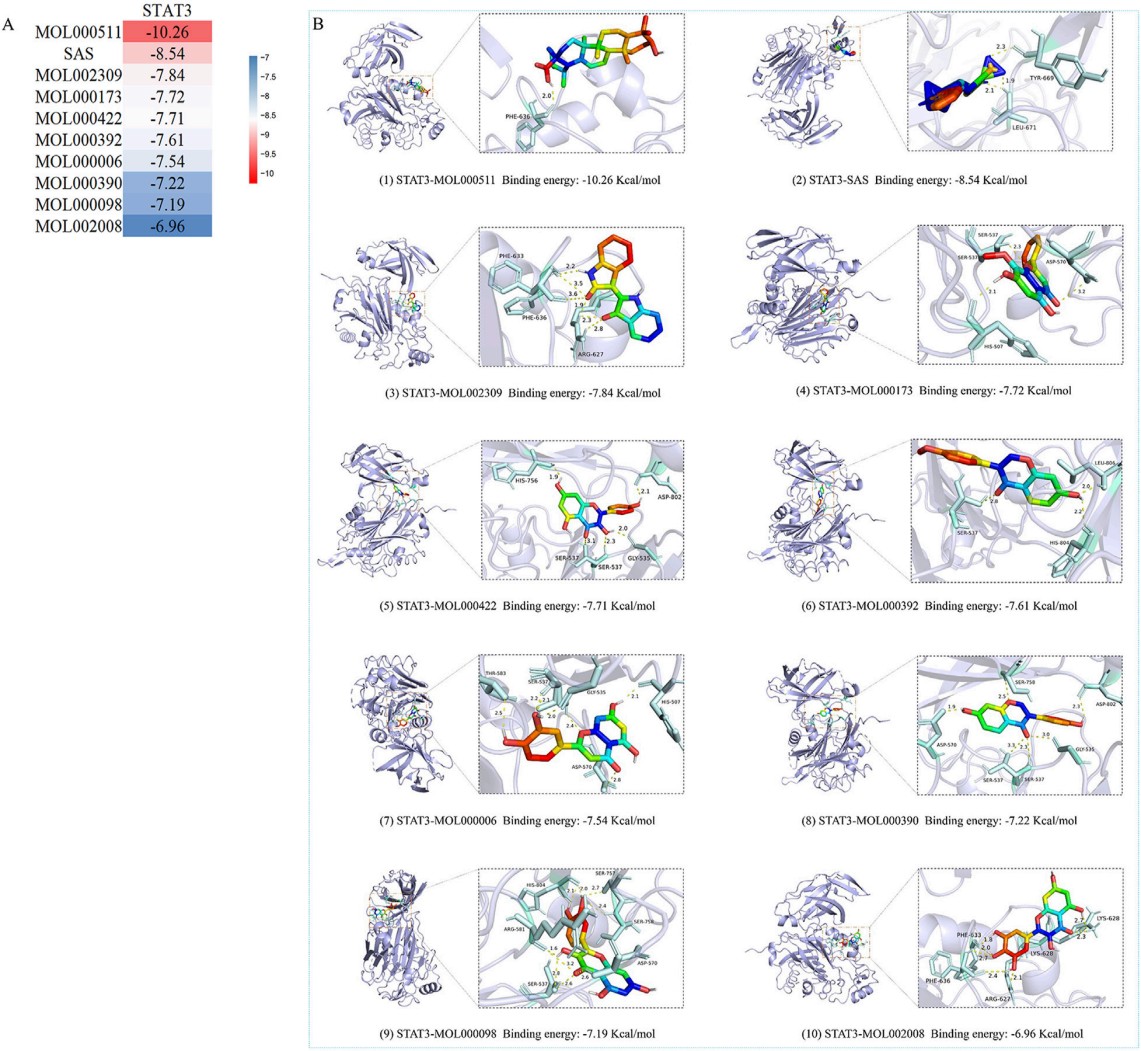
Molecular docking results. **(A)** Heatmap of the binding energies; **(B)** visualization of molecular docking results.

Based on PPI network analysis, we selected STAT3 as the core target. KEGG pathway analysis, revealed that STAT3 was enriched in Th17 cell differentiation, the JAK-STAT signaling pathway and the inflammatory bowel disease pathway; therefore, we selected STAT3 as the core target for molecular docking with the top 10 chemical components, Figure 5A. Subsequent animal experiments to investigate the IL6/JAK2/STAT3 pathway and Th17 cell differentiation were also carried out. The 10 selected chemical components included the component of SASP (C18H14N4O5S) and 9 components of YHKJ (quercetin, luteolin, ursolic acid, daidzein, kaempferol, wogonin, myricetin, formononetin, indirubin). STAT3 was molecularly docked with SASP and 9 YHKJ chemical components, and the docking results are presented in the form of a thermal map. These 10 sets of docking results were visualized and analyzed, Figure 5B. The related amino acids (AA) of these 10 chemical components can interact with STAT3 through hydrogen bonds: ursolic acid (AA: PHE), indirubin (AA: ARG/PHE), kaempferol (AA: GLY/SER/HIS/ASP), formononetin (AA: SER/LEU/HIS), daidzein (AA: SER/ASP/GLY), quercetin (AA: SER/ASP/ARG/HIS), myricetin (AA: ARG/LYS/PHE), wogonin (AA: HIS/SER/ASP), luteolin (AA: HIS/GLY/SER/ASP/THR) and SASP(AA: LEU/TYR). Moreover, myricetin, wogonin, luteolin and SASP can also interact with STAT3 through hydrophobic interactions: myricetin (AA: LYS), wogonin (AA: HIS/ASP), luteolin (AA: HIS/SER/ASP) and SASP (AA: LEU). The lower the docking score of the pharmaceutical chemical components with the key target protein, the stronger the binding. When the docking score is less than 1.2 kcal/mol, the docking effect is very strong (Shahnawaz Khan et al., 2020). The results showed that the binding energy of STAT3 (PDB: 6DLG) and ursolic acid (MOL000511), a component of YHKJ, was the highest (−10.26 kcal/mol), followed by that of SASP with a binding energy was −8.54 kcal/mol, suggesting that ursolic acid may be the key chemical component of YHKJ in the treatment of UC.

## 4 HPLC qualitative analysis of the main components of YHKJ

**FIGURE 6:**
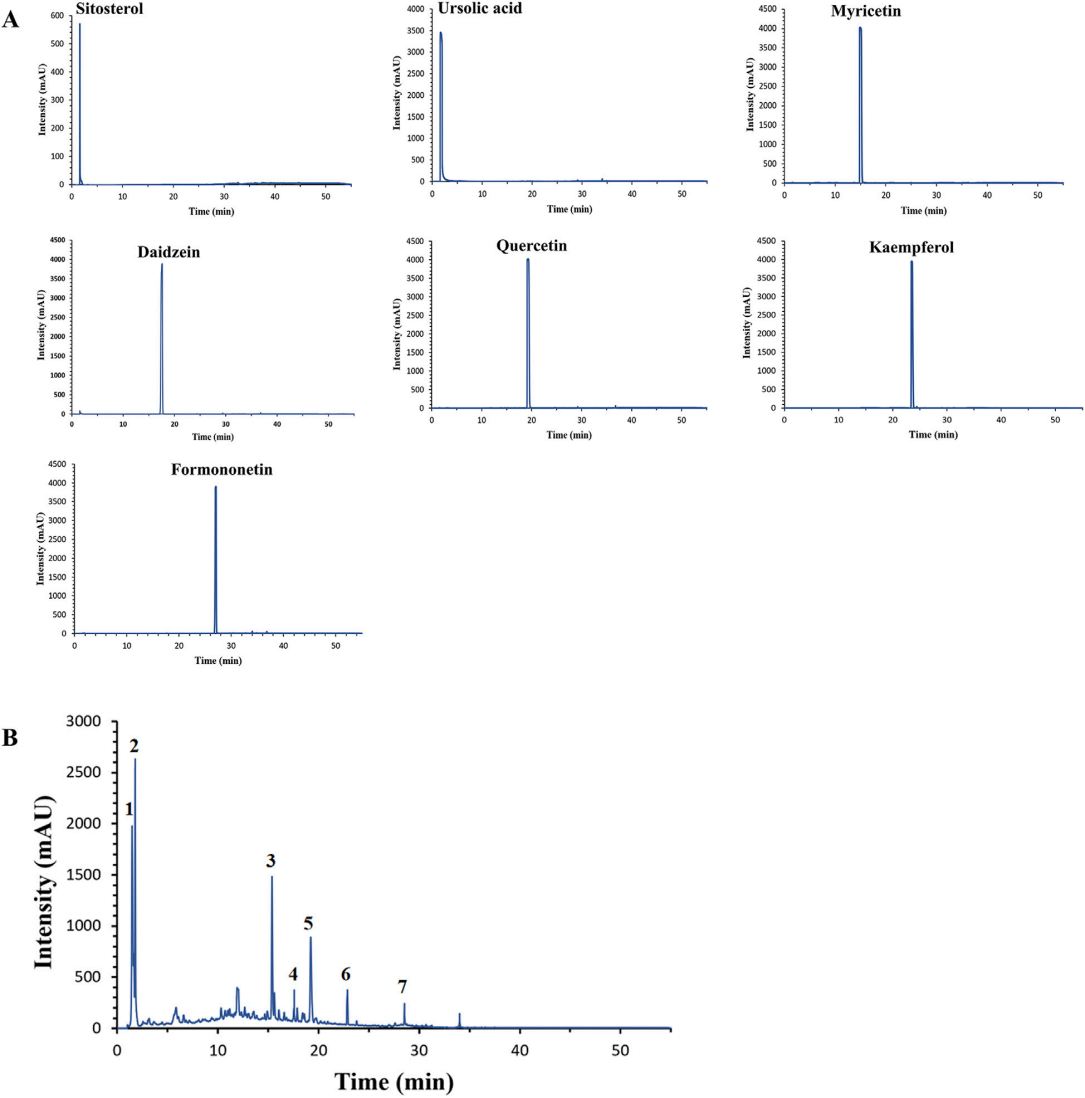
The elution times of the standard sample and the main components of YHKJ. **(A)** The elution times of the standard sample; **(B)** the elution times of the main components of YHKJ; 1 Sitosterol, 2 Ursolic acid, 3 Myricetin, 4 Daidzein, 5 Quercetin, 6 Kaempferol, 7 Formononetin.

HPLC analysis revealed that the elution times of the standard samples were as follows: sitosterol (1.593 min), ursolic acid (1.825 min), myricetin (15.176 min), daidzein (17.544 min), quercetin (19.224 min), kaempferol (23.555 min) and formononetin (23.555 min), Figure 6A. The main components of YHKJ included sitosterol, ursolic acid, myricetin, daidzein, quercetin, kaempferol and formononetin, and the elution times were the same as that of the standard sample, Figure 6B. These findings show that YHKJ may play a therapeutic role through these important compounds.

## 5 Experimental verification

### 5.1 HE staining was used to observe pathological changes in the colon tissue

**FIGURE 7:**
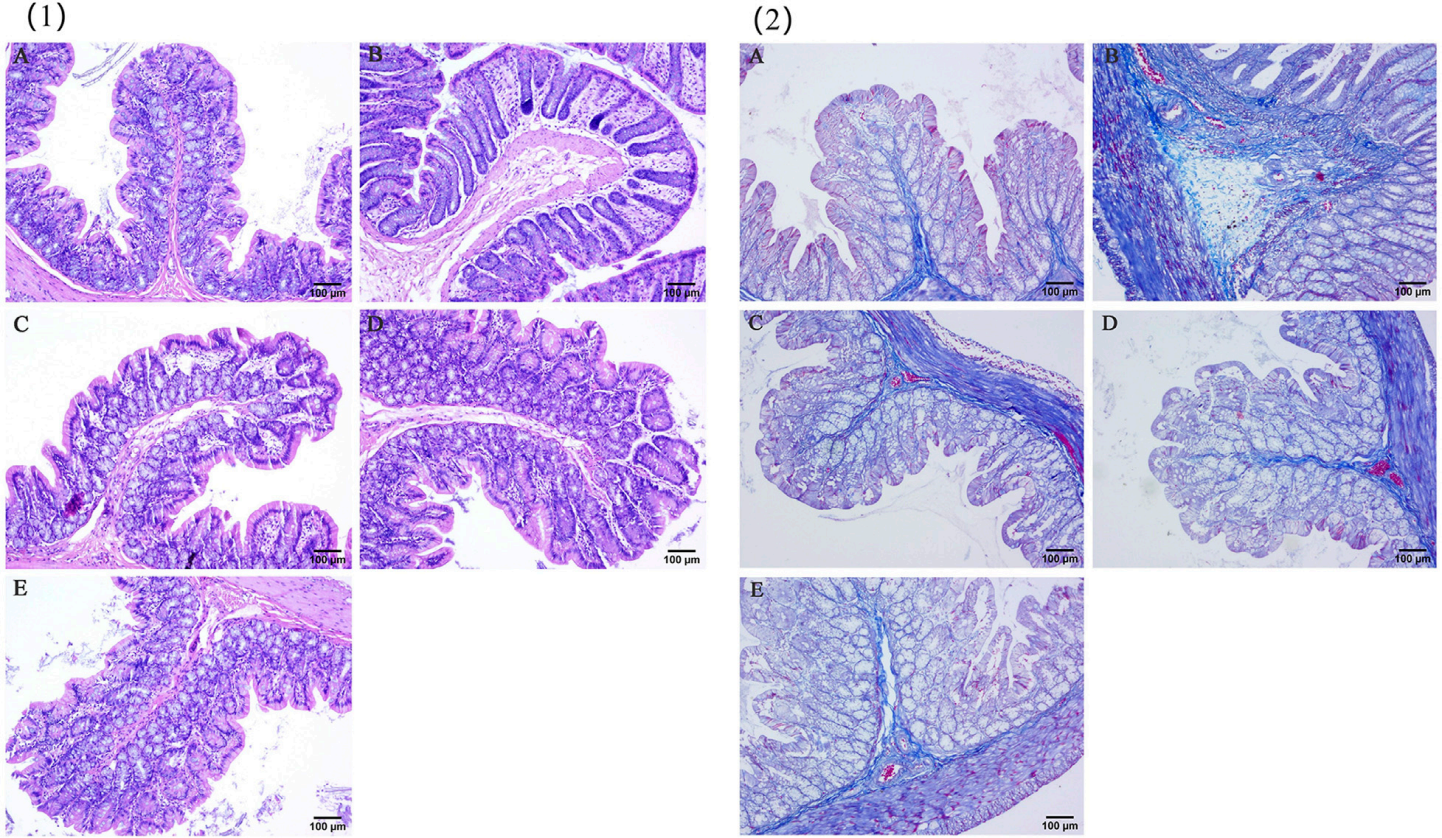
HE and Masson staining results. (1) HE staining results; (2) masson staining results; **(A)** Control, **(B)** Model, **(C)** YHKJF, **(D)** SASP, **(E)** YHKJF + SASP.

The pathological conditions of the colons of UC model rats in each group are shown in Figure 7. In the control group, the mucosa, submucosa, glands and crypts of the colon tissue were intact and continuous, and there was no edema, defects or faults. In the model group, inflammatory cell infiltration, edema of the mucosa and submucosa, increased recess spacing and some mucosal defects were discontinuous. The conditions of rats in the YHKJ group, the SASP group and the YHKJ + SASP group were significantly improved due to drug intervention. Edema in the mucosa and submucosa was reduced, but some mucosal glands in the YHKJ and SASP groups still exhibited edema, with slight mucosal defects and inflammatory cell infiltration. In the YHKJ + SASP group, the edema of the mucosa and submucosa was not obvious, the gland structure was compactly arranged, and the infiltration of inflammatory cells was reduced; these conditions are similar to those of the control group, except for some mucosal defects. Both YHKJ and SASP alleviated colonic lesions in UC model rats, and YHKJ combined with SASP led to the most significant improvement, Figure 7 (1).

### 5.2 Masson staining was used to observe changes in colonic fibrosis

In the control group, the intestinal mucosa was normal, and there was a small amount of collagen tissue in the submucosa. In the model group, a large amount of collagen hyperplasia was observed on the necrotic surface of the intestinal mucosa, submucosa, glandular space and basement. Compared with those in the control group, the amount of collagen tissue in the basal layer, submucosa and glandular space in the SASP group and the YHKJ group increased, but the extent of proliferation was reduced in the SASP group compared to that of model group. The proliferation of collagen tissue in the basal layer and submucosa in the SASP + YHKJ group was similar to that in the control group and lower than that in the model group, the SASP group and the YHKJ group, but some collagen tissue proliferation was still observed in the glandular space, Figure 7 (2).

### 5.3 The combination of YHKJ and SASP reduced the expression of the serum inflammatory factor IL-6 and increased the expression of the anti-inflammatory factor TGF-β1 in UC model rats

**FIGURE 8:**
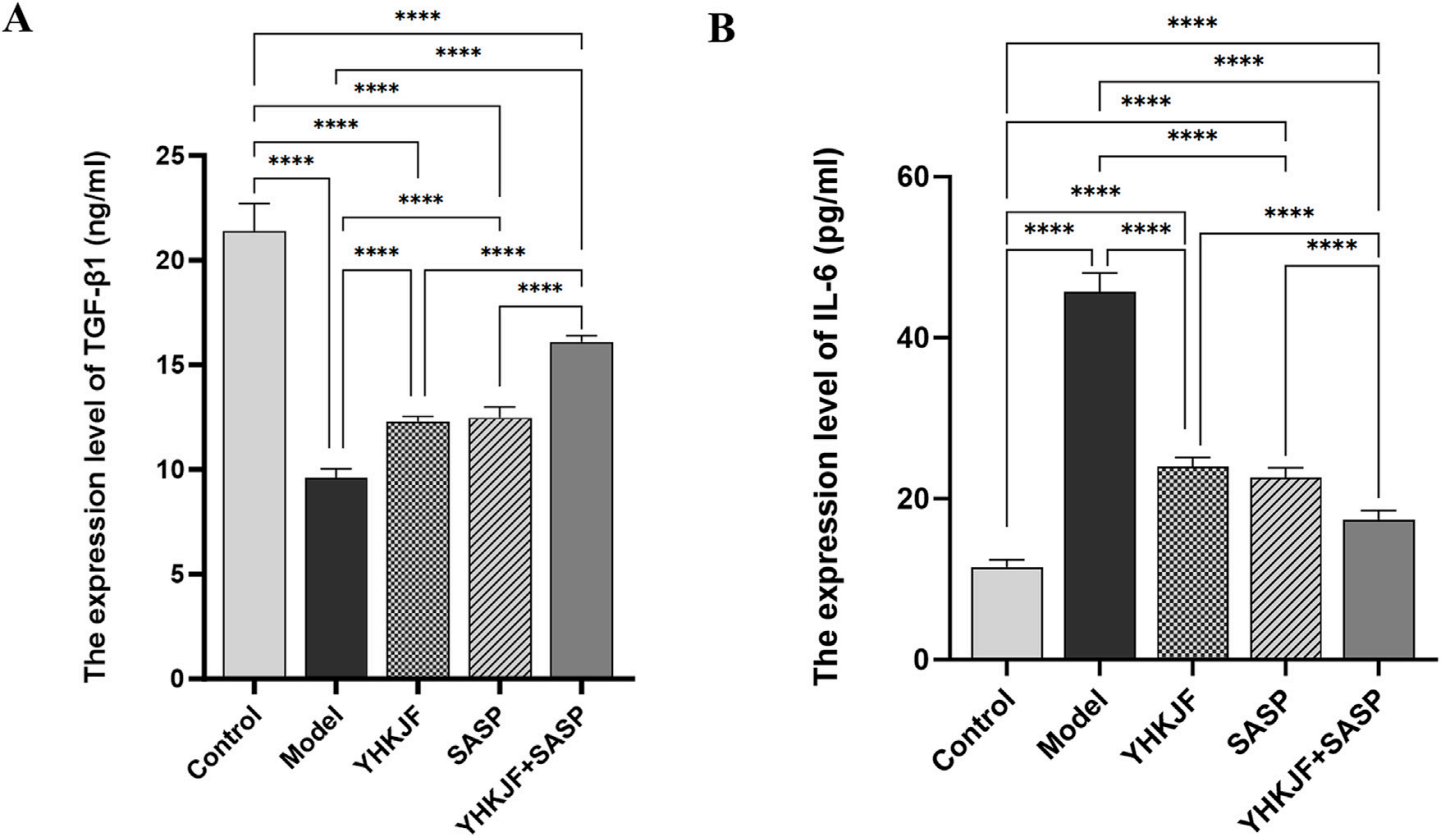
ELISA results of TGF-β1 and IL-6. **(A)** The expression results of TGF-β1; **(B)** the expression results of IL-6.

As shown in Figure 8, the expression of IL-6 in the serum of UC model rats increased significantly, while the expression of the anti-inflammatory factor TGF-β1 decreased; moreover, YHKJ and SASP treatment significantly decreased the expression of IL-6 and increased the expression of the anti-inflammatory factor TGF-β1 in the serum of UC model rats. Decreases in IL-6 and increases in TGF-β1 expression were observed in the YHKJ, SASP and YHKJ + SASP groups, especially in the YHKJ + SASP group. However, there was no significant difference in the expression of IL-6 or TGF-β1 between the YHKJ group and the SASP group, Figure 8.

### 5.4 The combination of YHKJ and SASP affects the relative mRNA levels of Treg/Th17-related factors in the colon tissue of UC model rats

**FIGURE 9:**
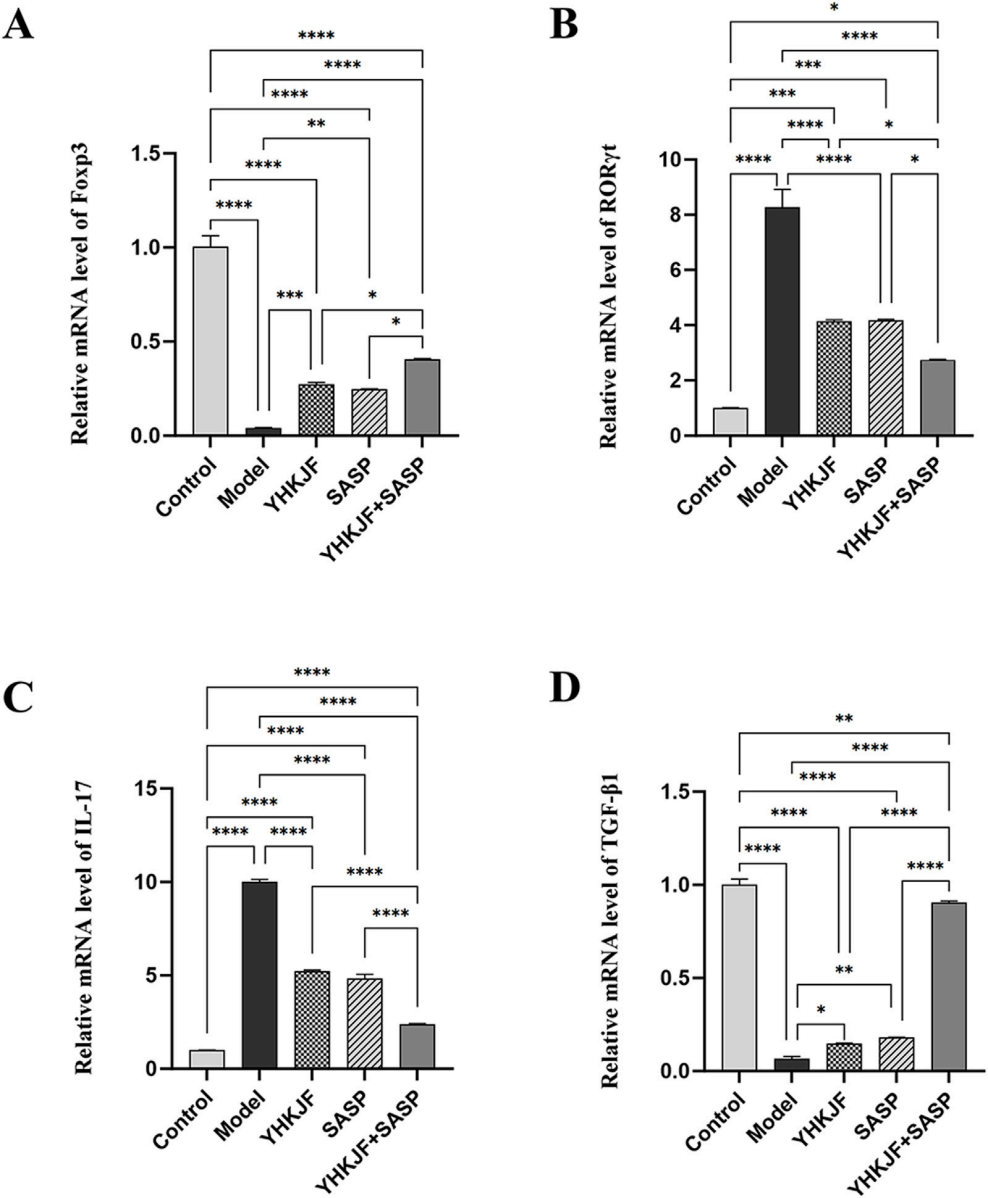
RT‒qPCR results of Foxp3, RORγt, IL-17 and TGF-β1. **(A)** Relative mRNA level of Foxp3; **(B)** relative mRNA level of RORγt; **(C)** relative mRNA level of IL-17; **(D)** relative mRNA level of TGF-β1.

RT‒qPCR analysis revealed that compared with those in the control group, the relative mRNA levels of IL‒17 and RORγt in the colon tissue of UC model rats in the model group increased, while the relative mRNA levels of TGFβ1 and Foxp3 decreased (Figure 9, *P* < 0.05). Treatment with YHKJ and SASP decreased the expression of IL-17 and RORγt, and increased the expression of TGFβ1 and Foxp3, especially in the YHKJ + SASP group. But there was no significant difference in the mRNA expression of IL-17, RORγt, TGFβ1 and Foxp3 between the YHKJ and SASP groups. Figure 9.

### 5.5 YHKJ combined with SASP reduced IL-6/JAK2/STAT3 signal transduction in the colon tissue of UC model rats

**FIGURE 10:**
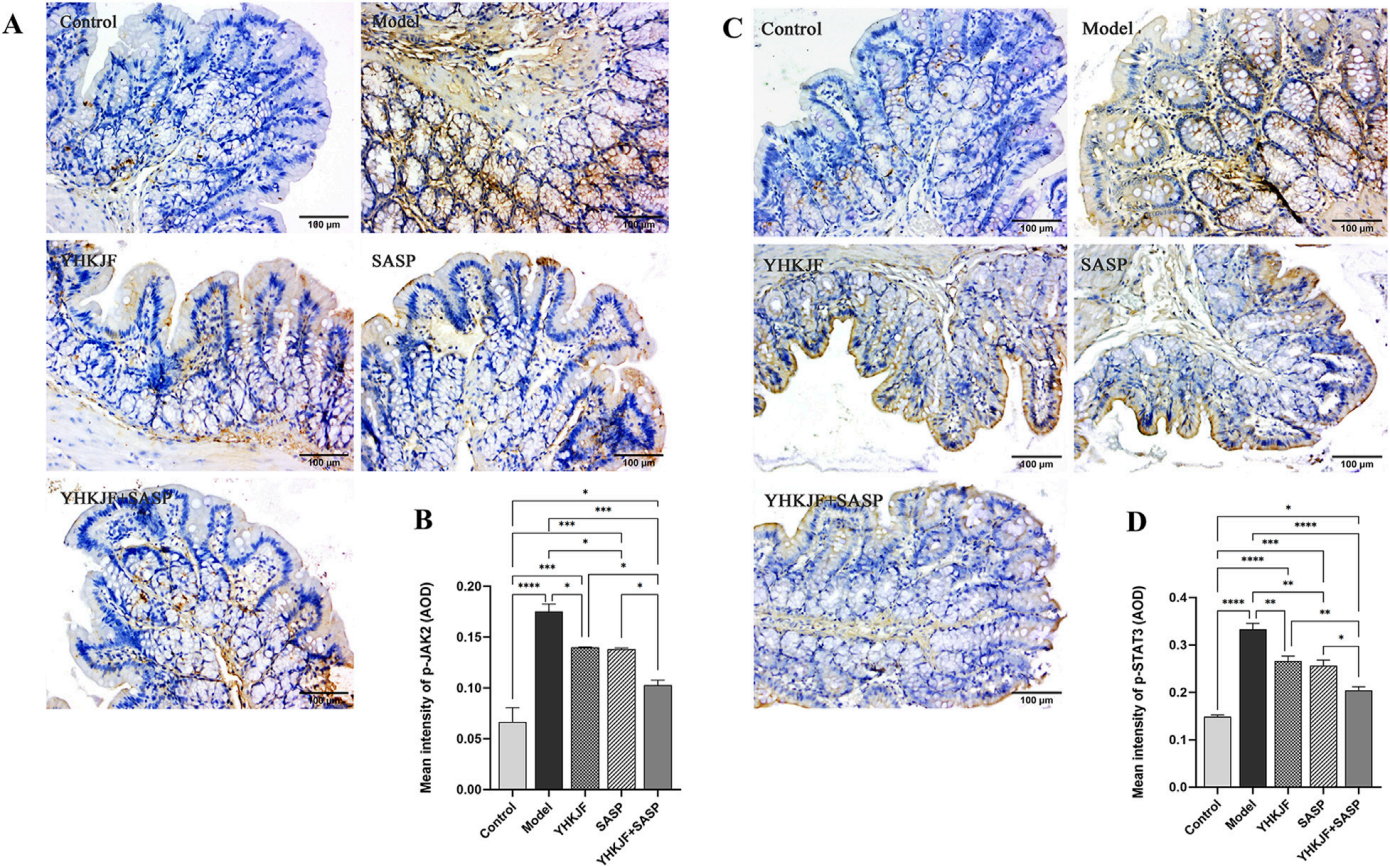
IHC results of p-JAK2 and p-STAT3. **(A)** IHC results of p-JAK2; **(B)** the expression results of p-JAK2; **(C)** IHC results of p-STAT3; **(D)** the expression results of p-STAT3.

IHC analysis revealed that the p-JAK2/p-STAT3 protein was mainly expressed in the mucosal and muscular layers of UC model rats, mainly in the mucosal layer. Compared with those in the control group, the amount of brown staining for p-JAK2 and p-STAT3 in the model group increased (Figure 10, *P* < 0.05). Compared with those in the model group, both the SASP and YHKJ groups exhibited reduced brown staining, and the brown staining of p-JAK2 and p-STAT3 in the YHKJ + SASP group decreased most significantly, but was still greater than that in the control group. However, the amount of brown staining for p-JAK2 and p-STAT3 in the YHKJ and the SASP groups was lower than that in the model group but still greater than that in the control group and the YHKJ + SASP group; there was no significant difference between the YHKJ group and the SASP group. Figure 10.

### 5.6 The combination of YHKJ and SASP reduced the relative protein expression of IL-6/JAK2/STAT3 in the colon tissue of UC model rats

**FIGURE 11:**
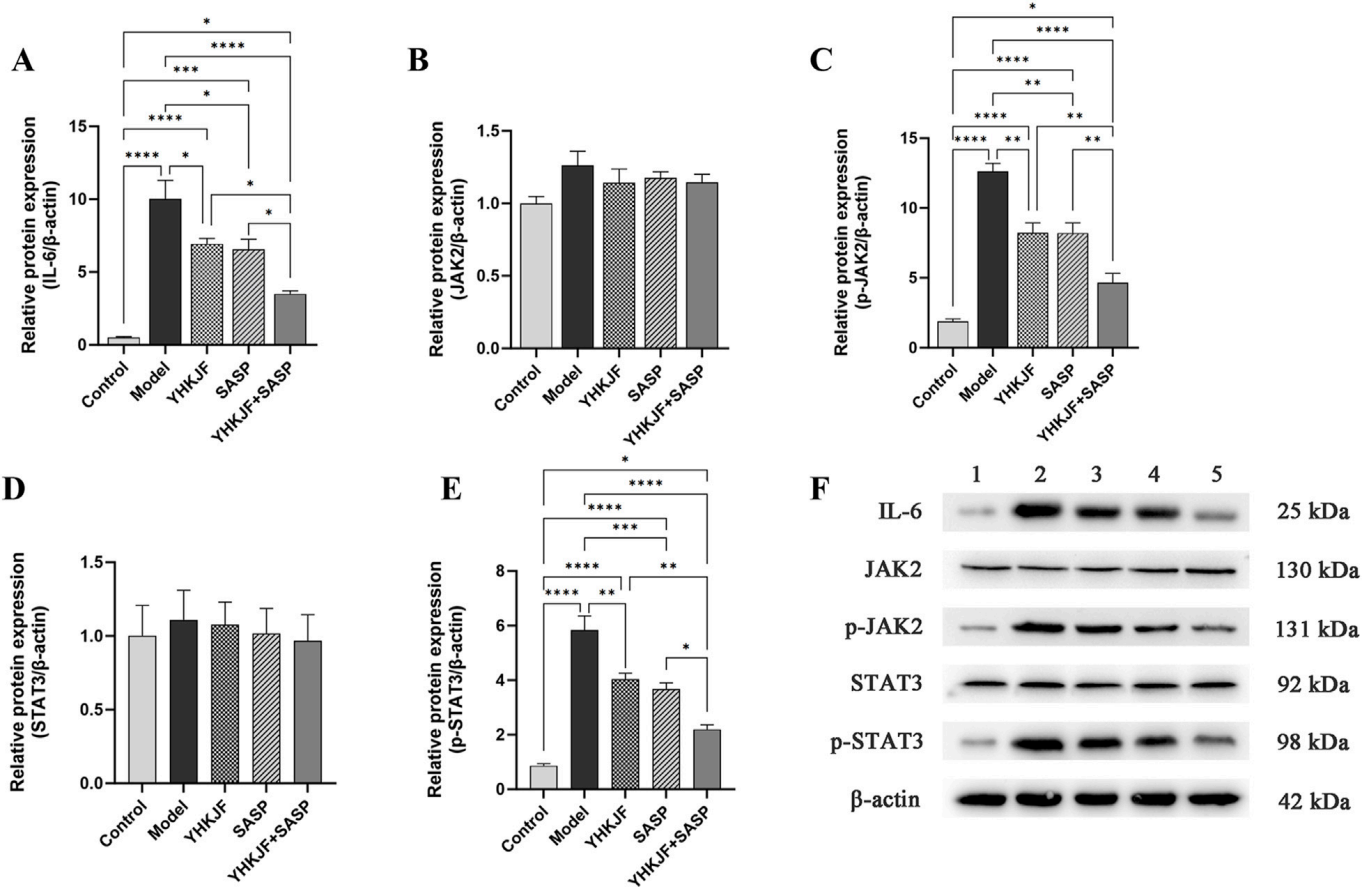
WB results of IL-6, JAK2, p-JAK2, STAT3 and p-STAT3. **(A)** Relative protein expression of IL-6; **(B)** relative protein expression of JAK2; **(C)** relative protein expression of p-JAK2; **(D)** relative protein expression of STAT3. **(E)** relative protein expression of p-STAT3; **(F)** the expression results of IL-6, JAK2, p-JAK2, STAT3 and p-STAT3.

Compared with other groups, the relative protein expression levels of IL-6, JAK2 and p-STAT3 in the model group increased (Figure 11, *P* < 0.05). The relative protein levels of IL-6, p-JAK2 and p-STAT3 in the YHKJ and SASP groups were lower than those in the model group but still greater than those in the control group and the YHKJ + SASP group. The decrease in IL-6, p-JAK2 and p-STAT3 in the YHKJ + SASP group was the most significant, but it was still greater than that in the normal group. There was no significant difference in the YHKJ and SASP groups. However, the relative protein expression of JAK2 and STAT3 did not change significantly among the groups, Figure 11.

## 6 Discussion

In recent years, the incidence and canceration rate of UC have obviously increased (Padoan et al., 2023). The etiology of UC is not clear, but an abnormal immune response and inflammatory response are thought to be important internal factors leading to inflammation and tissue damage in UC patients (Bharti and Bharti, 2022; Yuan et al., 2019). Therefore, researchers have focused on controlling the inflammatory immune response in UC patients and maintaining UC remission.

YHKJ, an in-hospital preparation that is used at the Third Affiliated Hospital of Liaoning University of Traditional Chinese Medicine, is an effective prescription for the treatment of UC in the clinic (Wang, 2023; Wang et al., 2022). Our project team has studied the mechanism of YHKJ in the treatment of UC in the early stage, and accumulated clinical research and basic experimental data provide the basis for this study (Li and Liu, 2024; Liu et al., 2012a; Liu et al., 2012b; Wang, 2023; Wang et al., 2022). Sulfasalazine, a 5-aminosalicylic acid, has the advantages of low cost, rapid onset and good curative effects. Research has shown that this medicine can regulate the immune function of UC patients and alleviate intestinal inflammatory reactions in UC patients(Mikami et al., 2023; Elhefnawy et al., 2023). TCM and Western medicines have their own advantages in the treatment of UC, so we used a combination of YHKJ combined with SASP in the treatment of UC and explored the advantages of integrating TCM and Western medicine in the treatment of UC.

Network pharmacology and molecular docking technology are auxiliary means to explore the mechanism of drug therapy for diseases, which can be used to systematically and comprehensively predict and analyze the chemical components of drugs and identify molecular mechanisms such as targets and pathways that act on diseases (Zhao et al., 2023; Sun et al., 2022; Shahzadi et al., 2024; Xiang et al., 2024). In this study, the chemical components and mechanism of the combination of YHKJ and SASP in the treatment of UC were investigated via network pharmacology and analytical docking methods. Considering the complexity of TCM components, we used HPLC to qualitatively analyze YHKJ components and identify the primary chemical components in YHKJ. The mechanism by which YHKJ combined with SASP in the treatment of UC was verified via animal experiments.

### 6.1 YHKJ combined with SASP in the treatment of UC has the advantages of overall regulation and local targeted treatment

Considering the complexity of TCM components, we carried out qualitative detection of YHKJ by HPLC to further determine the key chemical components. The HPLC results showed that YHKJ had seven main chemical components that played a role in relieving UC, namely, sitosterol, ursolic acid, myricetin, daidzein, quercetin, kaempferol and formononetin. Sitosterol, ursolic acid and myricetin account for a large proportion of the activity.

Sitosterol accounts for approximately 65% of herbal nutrition and can regulate immunity, resist microorganisms and treat colitis induced by dextran sulfate sodium (DSS) (Cheng et al., 2020; Pierre Luhata and Usuki, 2021; Zhang et al., 2023). Ursolic acid is the main component of some folk herbs and has antioxidative, anti-inflammatory, and immunomodulatory effects and protects intestinal function (Liu et al., 2016; Sheng et al., 2021; Shi et al., 2021; Wan et al., 2019; Wei et al., 2022). Myricetin is a flavonoid compound present in the bark, seeds and herbal leaves of Myrica rubra that can regulate immunity and apoptosis to prevent chronic UC (Duan et al., 2021; Zhao et al., 2022; Zhou et al., 2020). Daidzein is an isoflavone phytohormone that has anti-inflammatory effects and regulates intestinal function and the intestinal flora (Shen et al., 2019; Wu et al., 2020). Quercetin is also a flavonoid, that has anti-inflammatory and antioxidative functions and accelerates intestinal epithelial repair (Chittasupho et al., 2021; Liu et al., 2021; Sahoo et al., 2023). Kaempferol is a flavonoid in many medicinal plants that can resist inflammation, improve intestinal permeability and restore intestinal flora homeostasis (Kumar, 2020; Qu et al., 2021). Formononetin is also an isoflavone phytohormone that can alleviate colon inflammatory reactions and protect the intestinal mucosal barrier in UC model mice (Wu et al., 2018; Wu et al., 2020). These studies provide evidence that seven main YHKJ compounds have the potential to treat UC, and support the feasibility of our YHKJ research program.

SASP is an important drug for the targeted treatment of UC-related intestinal inflammation, which can be metabolized into 5-ASA and sulfapyridine in the colon. When 5-ASA is absorbed by intestinal epithelial cells, it can scavenge free oxygen produced by macrophages and neutrophils and regulate the migration of inflammatory cells, thus playing a targeted anti-inflammatory role (Mikami et al., 2023). Compared with simple Chinese medicine compounds, single medicines or synthetic medicines, the Chinese medicine YHKJ can treat UC through multiple targets and channels and has the characteristics of broad and extensive regulation. SASP, on the other hand, has the characteristics of accurate and exact local treatment (Mansuri et al., 2023; Youn et al., 2024). The combined use of YHKJ and SASP may better reveal the mechanism of synergistic drug therapy and improve the safety of drug use, which may be advantageous for overall regulation and local targeted therapy.

### 6.2 IL-6/JAK2/STAT3 participate in regulation of the immune inflammatory response in UC

As an important intracellular signal transduction pathway, the JAK2/STAT3 pathway is a key regulator of many biological processes, such as innate immunity, adaptive immunity and inflammation (Qin et al., 2022). JAK2/STAT3 is activated by the cytokine IL-6, which is related to the pathogenesis of UC (Duan et al., 2023; Yu et al., 2021). Existing evidence shows that IL-6 can be detected in the serum and diseased intestinal mucosa of UC patients, which is closely related to the inflammatory reaction, ulcer formation and severity of UC (Duan et al., 2023; Li et al., 2021; Ding et al., 2021). IL-6 is an important inflammatory mediator involved in the regulation of UC pathological processes and JAK2/STAT3 signaling pathway transduction (Duan et al., 2023; Nakase et al., 2022). IL-6 can regulate inflammation and the immune response, and downstream p-JAK2 can be activated by the inflammatory factor IL-6, thus further promoting the phosphorylation and activation of STAT3 (Chen et al., 2022). The severity of UC in patients is related to increased levels of phosphorylated STAT3 (Duan et al., 2023; Li et al., 2021). Blocking the JAK2/STAT3 pathway can regulate the immune response, thus dampening the intestinal inflammatory response in UC patients (Qin et al., 2022).

Moreover, IL-6 can also affect the proliferation and differentiation of T cells (Grivennikov et al., 2009; Qin et al., 2022). Both Th17 cells and regulatory T (Treg) cells are derived from CD4^+^ T cells, which can differentiate and develop under the control of the specific transcription factors Foxp3 and RORγt (Lee, 2018; Zhao et al., 2021). TGF-β can regulate the expression of Foxp3 and RORγt, which are especially important for the differentiation and development of Treg cells and Th17 cells (Larson et al., 2020; Xiao et al., 2024). A high concentration of TGF-β can increase the expression level of Foxp3 and induce CD4^+^ T cells to differentiate into Treg cells with anti-inflammatory effects, whereas a low concentration of TGF-β and a high concentration of IL-6 can increase the expression level of RORγt and induce CD4^+^ T cells to differentiate into Th17 cells, which promote the development of inflammation and produce characteristic IL-17A (Lv et al., 2023; Zhao et al., 2021).

The JAK2/STAT3 signaling pathway and Th17/Treg cells are involved in upstream and downstream regulatory relationships, whereas IL-6 can activate the JAK2/STAT3 signaling pathway and induce Th17 cells to differentiate and produce inflammatory reactions (Huang et al., 2019; Shan et al., 2023; Xia et al., 2022). When an intestinal inflammatory reaction occurs, Th17 cells can release IL-17A under the regulation of orphan nuclear receiver γt (RORγt), and further promote the release of inflammatory factors such as TNF-α and IL-6, which can amplify the inflammatory cascade and aggravate intestinal mucosal injury (Jia et al., 2024; Xiao et al., 2024). Therefore, downregulating the expression of the inflammatory factor IL-6, regulating activation of the JAK2/STAT3 signaling pathway, inhibiting the differentiation of Th17 cells and inhibiting the release of IL-17A are the keys to controlling the intestinal inflammatory response in UC patients, and maintaining intestinal immune homeostasis is the key to treating UC.

### 6.3 YHKJ combined with SASP inhibited immune inflammatory reactions through the IL-6/JAK2/STAT3 signaling pathway and relieved UC

To further clarify the mechanism of action of YHKJ combined with SASP in the treatment of UC, we performed KEGG network pharmacologyanalysis and found that STAT3, a core target, is involved in the pathological changes in UC in the inflammatory bowel disease pathway. Considering that STAT3 belongs to the JAK-STAT signaling pathway, the animal experiment results verified that the mechanism of action of YHKJ combined with SASP in the treatment of UC involves the IL-6/JAK2/STAT3 signaling pathway. Moreover, KEGG analysis revealed that STAT3, the core target, also participates in Th17 cell differentiation, and studies have shown that the JAK2/STAT3 signaling pathway can activate RORγt transcription and regulate Th17 cell differentiation and the release of inflammatory factors (Li et al., 2022). Therefore, we not only evaluated the mRNA expression of the anti-inflammatory and proinflammatory cytokines TGF-β1 and IL-17A in the colon mucosa by RT-qPCR, but also analyzed the mRNA expression of Th17 and the Treg cell markers RORγt and Foxp3 to preliminarily determine the influence of YHKJ combined with SASP on the JAK-STAT signaling pathway and the differentiation of downstream Th17/Treg cells.

**FIGURE 12:**
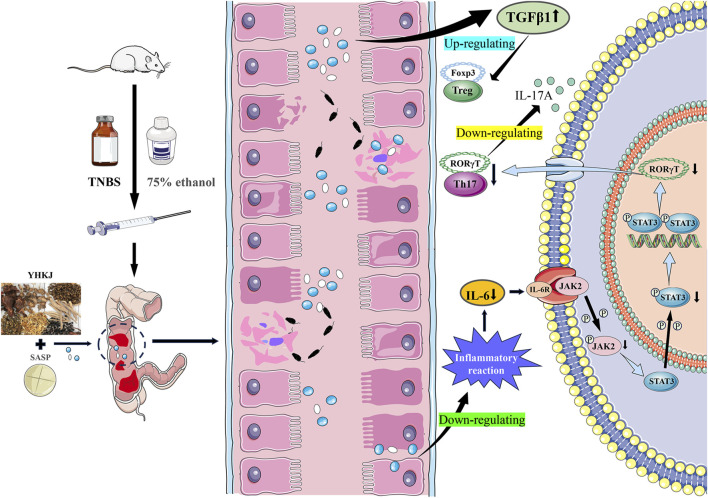
Molecular mechanism of YHKJ prescription intervening UC.

The results suggested that the YHKJ, SASP and YHKJ + SASP treatments downregulated the expression of the inflammatory factor IL-6 in the serum and the protein expression of IL-6, p-JAK2, and p-STAT3 and the mRNA expression of IL-17A and RoRγt in the colon tissue of UC model rats, and upregulated the mRNA expression of the anti-inflammatory factor TGF-β1 in the serum and of TGF-β1 and Foxp3 in the colon tissue of UC model rats. However, the regulatory effect in the YHKJ + SASP group was greater than that in the YHKJ group and SASP group, and there was no statistically significant difference between the YHKJ group and SASP group. These results indicate that, compared with the YHKJ group and SASP group, the YHKJ + SASP group presented downregulated protein expression of IL-6, p-JAK2 and p-STAT3 in UC model rats, thus further reducing the expression of the downstream proinflammatory factor IL-17A and Th17 cell marker RoRγt, and increasing the expression of the anti-inflammatory factor TGF-β1 and Treg cell marker Foxp3, thus playing an anti-inflammatory and therapeutic role in UC. Figure 12. YHKJ combined with SASP can alleviate UC through the IL-6/JAK2/STAT3 signaling pathway and regulate the differentiation of Th17/Treg cells to some extent, and this combination can be further studied from the perspective of Th17/Treg cell differentiation in the future.

Based on the above results, in order to further elucidate the mechanism by which the chemical composition of YHKJ treats UC through the IL-6/JAK2/STAT3 signaling pathway. The first nine chemical components of YHKJ with the strongest binding ability in the molecular docking results were detected by HPLC. The HPLC results revealed that YHKJ had therapeutic effects through seven main chemical components, including sitosterol, ursolic acid, myricetin, daidzein, quercetin, kaempferol and formononetin. These chemical components are not specific components of a single drug but are common to many traditional Chinese medicines. These findings indicate that YHKJ does not act through a single specific component but rather through a variety of compounds that act on STAT3 targets to treat UC, which shows that YHKJ has the advantage of multicomponent targeted regulation of STAT3 targets to treat UC. Many studies have shown that these seven chemical components play important roles in regulating the IL-6/JAK2/STAT3 signaling pathway. Among them, ursolic acid can not only inhibit the activation of the JNK/JAK/STAT signaling pathway in a Drosophila UC model and maintain the proliferation of intestinal stem cells to prevent intestinal mucosal injury, but also inhibit the expression of IL-17A and RORγt and the differentiation of Th17 cells, thus regulating immunity (Wang et al., 2023; Wei et al., 2022). Sitosterol can reduce the expression level of IL-6 in intestinal tissue (Ding et al., 2019). Myricetin can alleviate the symptoms of acute UC and upregulate the expression of TGF-β, and myricetin derivatives can inhibit the activation of the NF-kB/IL-6/STAT3 pathway (Duan et al., 2021; Zhao et al., 2022). Daidzein inhibited the expression of IL-6 and IL-1β in mice with DSS-induced colitis (Shen et al., 2019). Quercetin regulates the differentiation of Th17/Treg cells and inhibits the production of TNF-α, IL-1β and IL-6 (Riemschneider et al., 2021; Zhang Q. et al., 2022). Kaempferol can maintain intestinal integrity, reduce the expression of IL-6 and inhibit the activation of the JAK/STAT3 pathway (Bian et al., 2020; Qu et al., 2021; Zhang Z. et al., 2022). Formononetin can reduce the expression of IL-6 to alleviate colitis symptoms in mice and inhibit JAK2/STAT3 signaling pathway transduction to protect against cerebral ischemia reperfusion injury (Wu et al., 2018; Yu et al., 2022).

SASP is a standard therapeutic drug that can induce UC remission, which can improve the symptoms and pathological damage to the colon in DSS-induced UC model mice and has a regulatory effect on the IL-6/JAK2/STAT3 signaling pathway (Elhefnawy et al., 2023; Wu et al., 2022). SASP can reduce oxidative stress and the inflammatory response in TNBS-induced colitis model rats by reducing the expression of TNF-α and IL-1β and inhibiting IL-6/JAK2/STAT3 signal transduction (Elhefnawy et al., 2023). Another study revealed that SASP combined with Origanum Majorana L pretreatment can reduce the cascade of colon inflammation by inhibiting the IL-6 inflammatory factor and downregulating the JAK2/STAT3 signaling pathway and Th17 cell response, thus alleviating acetic acid-induced UC (Taha et al., 2023).

The above evidence shows that the SASP and the main components of YHKJ can inhibit the signal transduction of IL-6 or JAK2/STAT3, which is consistent with the results of this study. Our research is based on the treatment concept of integrated TCM and Western medicine and analyzes the mechanism of YHKJ combined with the SASP in the treatment of UC. The core targets and key signaling pathways of YHKJ combined with SASP in the treatment of UC were screened via network pharmacology and molecular docking, and the chemical components of YHKJ in the treatment of UC were identified by HPLC. The molecular mechanism of YHKJ combined with the SASP in the treatment of UC through the IL-6/JAK2/STAT3 signaling pathway was verified through animal experiments. Compared with the single use of SASP or YHKJ in the treatment of UC, the combined use of SASP and YHKJ can not only alleviate the pathological changes and fibrosis of colon tissue in UC model rats but also regulate the IL-6/JAK2/STAT3 signaling pathway through multiple components to treat UC. These findings suggest that the combined application of YHKJ and SASP can better reveal the mechanism of synergistic drug therapy, which is beneficial for overall regulation and local targeted therapy and has advantages in treating UC.

These findings suggest that, compared with simple Chinese medicine compounds and single medicines, the combination of YHKJ and SASP has the advantages of extensive regulation and multicomponent targeted therapy of TCM and local targeted anti-inflammatory effects of Western medicine, which is beneficial for overall regulation and local targeted therapy, thus revealing the mechanism of synergistic drug therapy and improving pharmacological efficacy. Our research provides new ideas for analyzing the complex mechanism of treating UC with integrated TCM and western medicine and provides a reference for the strategy of treating UC with integrated TCM and western medicine. However, the design of this study still needs to be improved. This study is based on previous research, so we chose the SASP as the intervention method. Although the SASP can effectively treat UC, many safety problems exist (Akao et al., 2024; Kuchinskaya et al., 2023). Therefore, with respect to the choice of Western medicine in follow-up studies, we will consider the use of mesalazine as a drug to intervene in UC to improve the safety of the medication.

## 7 Conclusion

In summary, in this study, we predicted the mechanism and key active components of YHKJ combined with SASP in the treatment of UC through network pharmacology and molecular docking. We qualitatively analyzed the main components of YHKJ by HPLC and found that YHKJ has seven main chemical components that play a role in relieving UC, namely, sitosterol, ursolic acid, myricetin, daidzein, quercetin, kaempferol and formononetin. The docking results showed that the binding energy of the chemical component ursolic acid in YHKJ to STAT3 was the highest (−10.26 kcal/mol), followed by that of SASP (C18H14N4O5S) with a binding energy was −8.54 kcal/mol, suggesting that ursolic acid may be the key chemical component of YHKJ in the treatment of UC. According to the key PPI targets and KEGG pathway enrichment analysis results, STAT3 had the strongest correlation with the pathway of action nd participated not only in the pathological changes in UC in the inflammatory bowel disease pathway but also in Th17 cell differentiation and the JAK-STAT signaling pathway. Considering that STAT3 is involved in the JAK-STAT signaling pathway, in our study, we verified that YHKJ combined with SASP inhibits activation of the IL-6/JAK2/STAT3 signaling pathway, reduces intestinal inflammation and relieves UC. Our research may provide new insights for analyzing the complex mechanism of TCM combined with Western medicine in the treatment of UC and may provide a new reference for treatment strategies involving integrations of TCM and Western medicine for UC.

## Data Availability

The raw data supporting the conclusions of this article will be made available by the authors, without undue reservation.
