# Supplementary figures and images for: Combination of Youhua Kuijie Prescription and sulfasalazine can alleviate experimental colitis via IL-6/JAK2/STAT3 pathway
Source: Front Pharmacol. 2024 Sep 10;15:1437503. doi: 10.3389/fphar.2024.1437503 (PMC11420560; doi:10.3389/fphar.2024.1437503)

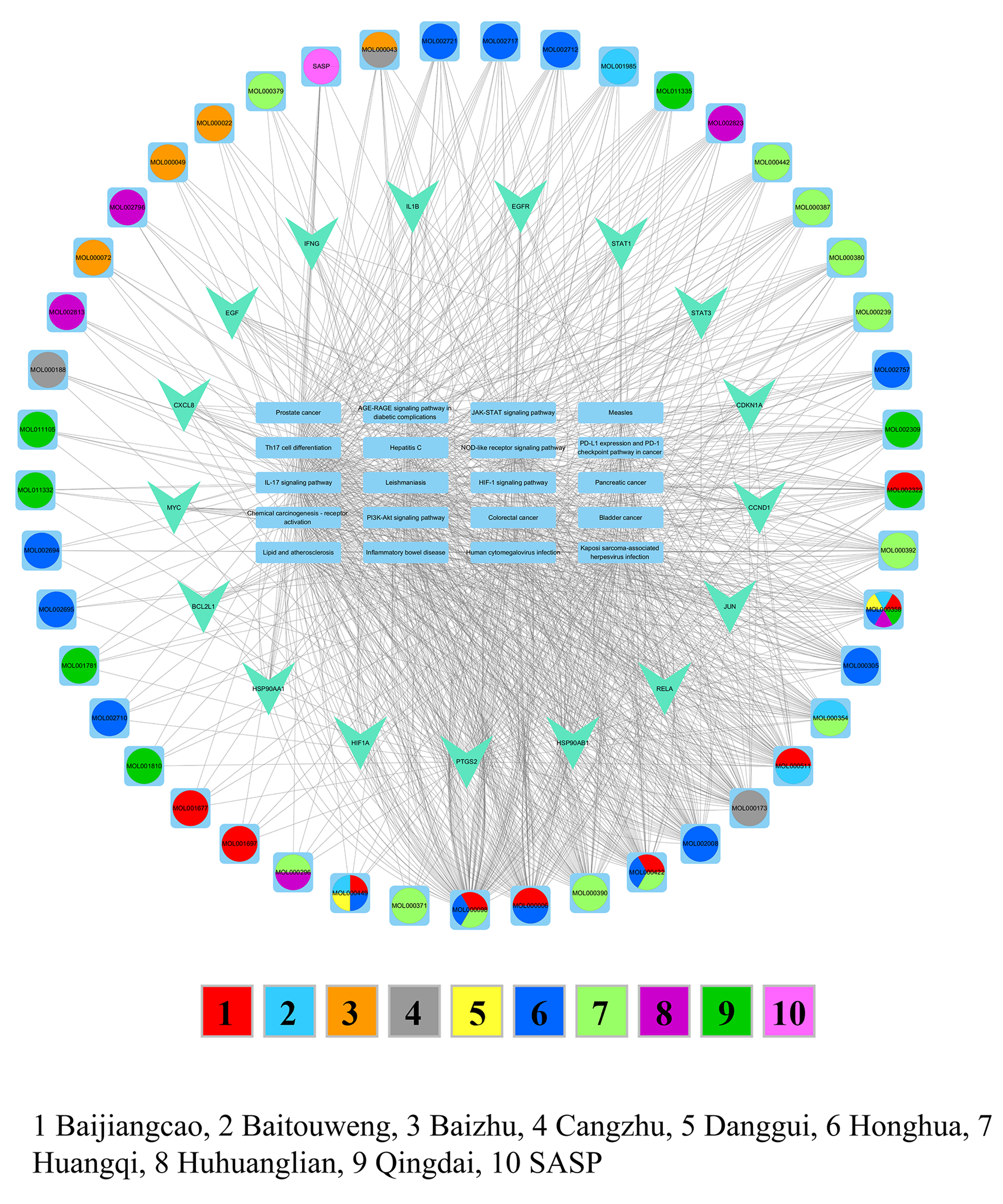

Supplement: Supplementary file 1 [file Image2.tif]

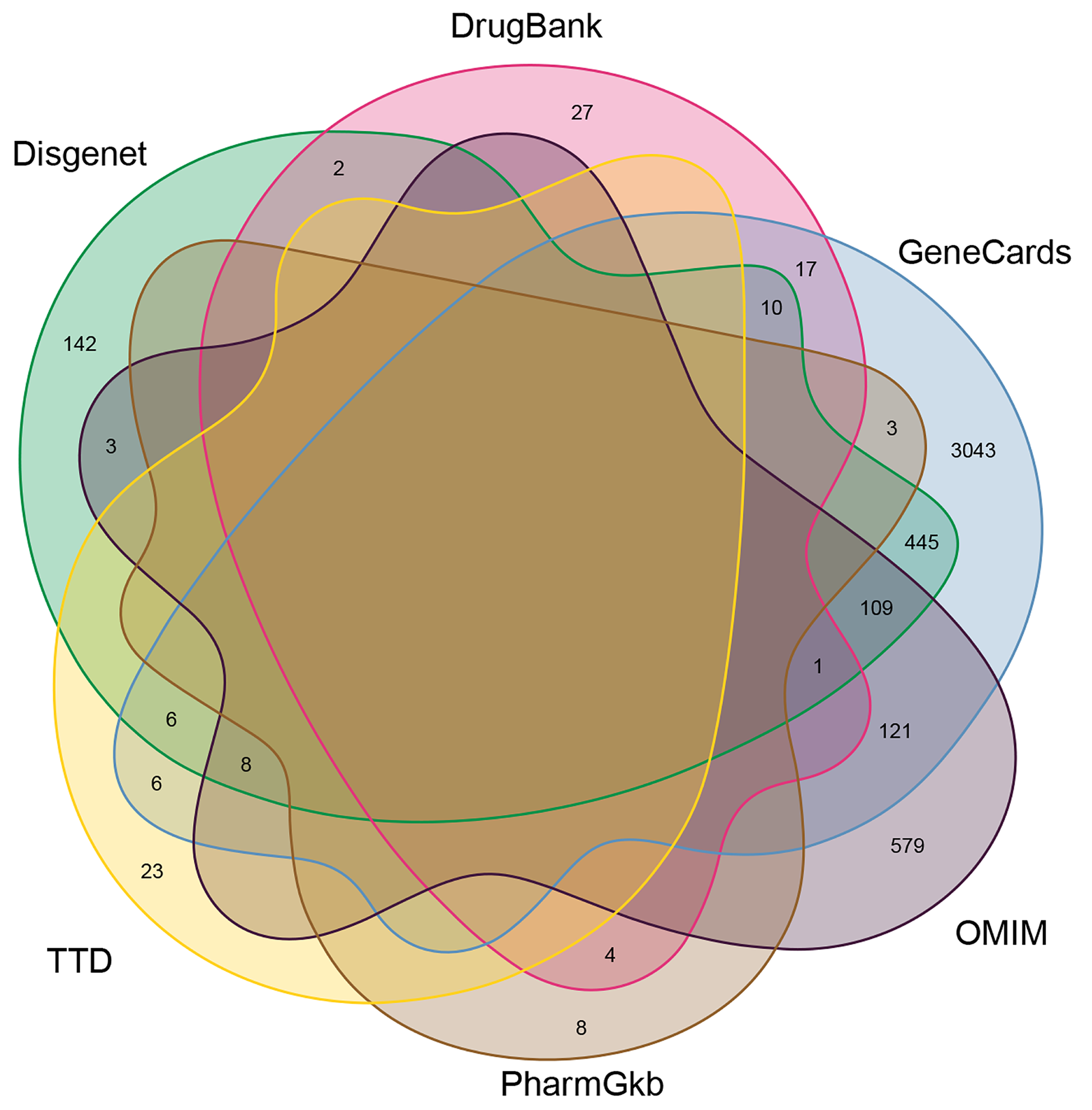

Supplement: Supplementary file 2 [file Image1.tif]
